# Reliability and validity of simple Chinese version of grit scale for elementary school students

**DOI:** 10.1186/s40359-024-01809-3

**Published:** 2024-05-28

**Authors:** Wang Jie, Wang Xinyi, Xin Tao

**Affiliations:** 1 College of Education for the Future, Beijing Normal University, Zhuhai519000, China; 2 Collaborative Innovation Center of Assessment toward Basic Education Quality, Beijing Normal University, Beijing100875, China

**Keywords:** Grit, Elementary School Students, Reliability, Validity

## Abstract

**Background:**

The Grit scale (GS-12) is a widely used rating scale that assess passion and perseverance. The present study aimed to evaluate the reliability and validity of simple Chinese Version of Grit Scale (GS-SC) among Chinese adolescents.

**Methods:**

Seven hundred one primary school students were recruited as Sample 1. Item analysis and exploratory factor analysis (EFA) were conducted on Sample 1 to preliminarily examine the structure of the scale. Sample 2 consisted of 5,384 primary school students. Confirmatory factor analysis (CFA) and verification of reliability and validity were conducted on Sample 2 to establish a formal scale and further verify the psychometric properties by applying item response theory (IRT).

**Results:**

EFA and CFA revealed a clear two-factor structure. The results demonstrated that the Simplified Chinese Version of Grit Scale had adequate internal consistency and re-test reliability. GS-CS also showed good criterion-validity with personality, self-control, effort regulation and academic achievement. Furthermore, all the items show a acceptable fit to the GRM and have good discrimination (ranging from 2.13 to 3.45) and moderate difficulty(ranging from-1.58 to 0.95).

**Conclusions:**

The reliability and validity of the GS-SC are good, indicating that the scale can be used as an effective tool for measuring the grit of primary school students in China.

## Background

It has been widely held that intellectual factors are the primary determinants of an individual’s success. However, over the past two decades, several scholars have argued that success is mostly determined by a trait called grit, which is significantly more important than intelligence [1]. By examining specific examples of remarkable achievements, Galton argued that self-denial and zeal were the primary causes of individual outstanding achievements [2]. According to Duckworth, Peterson, Matthews and Kelly [3], self-control alone, which helps individuals resist temptation and stay on track with their goals, is insufficient for achieving long-term goals. In addition to overcoming obstacles, it also requires passion to attain one’s ambitions. Consequently, researchers combined these two traits and named them ‘grit,’ and they developed a corresponding scale to measure it.

The concept of grit was defined as having passion and perseverance for long-term goals [3, 4]. It is proposed that there are two factors that characterize grit: Consistency of Interest (CI) and Perseverance of Effort (PE) [3]. Consistency of interest encompasses constantly adhering to interests or activities that can bring about achievement of long-term ambitions [5]. Perseverance of effort refers to the ability to endure and effectively manage setbacks and failures in pursuit of long-term goals [5].

Previous studies showed that grit was an important predictor variable of an individual’s success in various domains such as academic achievement [6–9], literacy achievement [10], physical activity [11], vocabulary knowledge [12], and sports [13], as well as positive educational and psychological functioning such as life satisfaction [7], mindfulness [14], personality(e.g. agreeableness) [3, 15–19], resilience [16, 20–22], self-control [15–18, 23], test emotion [24], and well-being [22, 25, 26]. Although there have been many studies on grit in the past, the results of a meta-analysis revealed that variables related to success, performance, and personality are the most studied [27]. Duckworth and colleagues [3] examined the importance of grit to achievement and discovered that grit accounted for an average of 4% of the variance in success outcomes, and was highly related to the personality trait of Big Five Conscientiousness but associate negatively with IQ. Moreover, Tovar-García [28] compared grit levels between migrants and native students in explaining school achievements and the findings revealed that grit had positive effects in explaining the educational achievements of migrant groups. Furthermore, Danner, Lechner and Rammstedt [29] investigated the relationship among grit, objective (income), subjective (job satisfaction) career success, cognitive skills and educational attainment. They found that associations between grit and career success could vary across individuals and contexts. Meanwhile, although personality significantly predicts academic achievement, grit adds little phenotypically or genetically to the prediction of academic achievement beyond traditional personality factors, especially conscientiousness [23]. These conflicting findings suggest that past research on grit and success is not entirely conclusive.

Personality is one of the variables that are highly related with grit [15–18]. Among the Big Five personality traits, grit has the strongest correlation with conscientiousness, showing that the more conscientious an individual is, the more resilient they are. Additionally, previous studies suggest that self-control is also commonly used criterion variable for assessing validity of the grit scale [3, 15–18], because self-control and grit share similar concepts [17]. Interestingly, the relations between grit and demographic variables such as gender was very weak [27]. There is no discernible difference in grit between gender, according to some prior studies [17, 30].

Besides, previous researches have also found that perseverance of effort and consistency of interests have exhibit different relationships with various indicators of academic achievement, positive psychological functioning, and work-related functioning. Perseverance of effort has been found to positively predict academic achievement [17, 31]. A meta-analysis showed that academic achievement is more strongly correlated with perseverance of effort (*ρ* = 0.26) than with consistency of interest (*ρ* = 0.10) [27]. The meta-analysis also showed that grit is moderately correlated with performance and retention, and strongly correlated with conscientiousness [27]. In addition, previous studies also showed that, perseverance of effort demonstrated a higher correlation than consistency of interest in predicting academic achievement [32–34]. Besides, mastery goals, performance-approach goals, and intrinsic motivation are all positively related to PE, whereas performance-approach, performance-avoidance goals, and extrinsic motivation are all negatively correlated with CI [35]. These findings indicate that PE and CI played different roles in achieving various goals.

In summary, as grit is a critical trait to personal growth, a high reliability and validity scale is required to evaluate it. It will provide reliable quantitative results and provide a solid foundation for further related studies. There are numerous types of scales and questionnaires available to assess grit among children, adolescents and adults. The most widely used assessments of grit are the Grit Scale (Grit-O) [3] and the Short Grit Scale(Grit-S) [36]. The Grit-O, which consists of 12 items and has two dimensions, was developed by Duckworth and colleagues [3], who also proposed the original theoretical model of grit. Although the structure of Grit–O was congruent with the theory of grit, it was found that the model fit of the Grit–O need improvement [36]. Duckworth and Quinn [36] later removed 4 items from the Grit–O and developed a validated 8-item Grit Short Scale (Grit-S), which retains the 2-component structure of the Grit–O. It was observed that the Grit–S was both shorter and psychometrically stronger than the Grit–O [36]. Additionally, some researchers have developed a series of scales for their own research purposes [7, 37, 38]. Kuruveettissery, Gupta and Rajan [37] combined the theoretical model of grit with goal-directed resilient responses, and developed a 17-item Grit Scale with three factors: Perseverance-Commitment (PC), Interest-Passion (IP), and Goal-directed Resilience (GR). In addition, based on the Grit-O, Sturman and Zappala-Piemme [38] defined grit as “To sustain a focused effort to achieve success in a task, regardless of the challenges that present themselves, and the ability to overcome setbacks” and they developed a new 12-item grit scale that would capture this definition and could be readable by schoolchildren and adults alike. In order to explore the relationship between grit and achievement, Clark and Malecki [7] developed a new academic grit scale which positively correlates with academic functioning and life/school satisfaction.

Although modern researchers have created a number of other grit scales, the Grit-O is still the most commonly used. There are also some researchers have translated the Grit-O into Chinese. For example, Taiwan researchers modified the Grit-O based on elementary school students [17]. However, the empirical research has pointed to the possibility of the misinterpretation of one item in the Grit-O, “New ideas and new projects sometimes distract me from previous ones,” among Chinese elementary school students [17]. After deleting the item 2, they developed a Traditional Chinese version of the Grit Scale (GS-TC) by modifying the sentences based on language skills of elementary school students.

However, to date, there is a dearth of grit scales designed specifically for primary school students in mainland China. It is believed that this is a crucial gap in the literature that need to be addressed. Previous research has demonstrated that grit is a critical personality trait that exerts a significant influence on students’ academic achievement and psychological well-being [7, 26]. Given that primary school is a critical period for children’s personality development [39], it is essential to cultivate and enhance students’ grit during this time. However, such efforts are predicated upon the availability of accurate assessment tools.

Although there is a traditional Chinese version of the grit scale has been revised based on data from primary school students in Taiwan, differences between mainland China and Taiwan in vocabulary and language use [40] suggest that the traditional Chinese version of the grit scale may not be suitable for mainland Chinese students. Moreover, although simplified Chinese versions of the grit scale has been developed for university students and adults, research has shown that individual grit levels change over time [3], and standards for grit performance vary with age and environmental context. Consequently, descriptions and standards related to grit traits in existing scales may not be applicable to children.

In terms of the grit scale’s psychometric qualities, the previously stated investigations mostly employed classical test theory (CTT) techniques [3, 17]. In CTT, an individual’s overall score on the scale items indicates where they fall in the construct. This approach has certain drawbacks even if it is simple to understand. The primary shortcomings of CTT include an over-reliance on samples, a failure to account for measurement error, a failure to consider the difficulty and discrimination of test items, a lack of adaptability to non-homogeneous testing, and an inability to directly estimate important test item parameters like discrimination and difficulty. In addition, Item Response Theory (IRT) is better suited for non-homogeneous and non-standardized tests, allowing for flexible handling of different test structures and item designs. Furthermore, IRT offers continuous estimation of the latent traits being measured, enabling a more accurate capture of the ability or trait level of the examinee. Direct estimation of parameters such as difficulty, discrimination, and slope of measurement items can provide a more precise description of measurement characteristics. The grit scale is not frequently used with students in primary schools, and it does not feature any items that have been validated through IRT.

This article’s goal is to show how, in situations when somewhat high sample sizes are feasible, CTT and MIRT can be used to assess the psychometric qualities of the GS-TC. Thus, the following are the questions that this study seeks to answer: 1) Does the GS-TC still have a two-dimensional structure among Chinese Mainland primary school students? If so, do the scale’s items need to be revised? What about the scale’s validity and reliability? 2)Is there a relationship between the scale and academic success, personality, effort regulation, and self-control? 3) Do the scale’s items fit the multidimensional item response model? If so, What about the scale’s psychological properties based on MIRT?

## Materials and methods

### Samples

We recruited sample 1 from seven schools in Jinhua and Taizhou for the purpose of pre-testing. This phase involved 701 students, with females being slightly more represented than males (50.4% vs. 49.4%). Sample 2 was comprised of 6013 participants in grade 4 who were recruited from 75 schools in Zhejiang province in China. After removing participants with missing data for the relevant study variables, the final sample consisted of 5384 students from 75 schools. Of these, 2896 (53.8%) were boys, and 2476 (46%) were girls. Sample 3 was recruited from a class in the city of Taizhou and consisted of 42 children (54.8% boys and 45.2% girls). Sample 3 were recruited to conduct the test-retest reliability analysis. All three studies were approved by the local research ethics committee.

### Procedure

The research team recruited a translator to translate the scale from traditional Chinese to simplified Chinese, and then back-translated from simple Chinese to English by the other translator.

Systematic sampling was used to collect data. Firstly, 70 elementary schools in Zhejiang province were selected. Secondly, students in these schools were sequenced by their last name and was selected into the present study at specific intervals. Then, with guidance from two teachers in each school, the students completed the GS-SC and other scales individually. Students were told that the survey was being used only for academic research, and that their privacy would be guaranteed. Additionally, students were instructed to select one option for each item based on their factual experience.

### Instruments

#### Simplified Chinese version of grit scale

The Grit Scale (GS) was first developed by Duckworth and Peterson [3]. It is a 12-item self-report scale and has two dimensions: perseverance of effort (6 items) and consistency of interest (6 items). Responses are categorized into on a five-point Likert scale ranging from 1 (*strongly disagree*) to 5 (*strongly agree*). The GS was later modified to the GS-TC in order to assess elementary school students in Taiwan [17]. The GS-TC is an 11-item scale with two dimensions: perseverance of effort (item 1, 4, 6, 9, 10, 12) and consistency of interest (item 3, 5, 7, 8, 11) [17]. The average coefficient 𝛼 were 0.78 and 0.71 for perseverance of effort and consistency of interest, respectively [17]. The test-retest coefficient of perseverance of effort was 0.64 and the test-retest coefficient consistency of interest of 0.52 [17]. We further modified the GS-TC into the GS-SC. The higher the score on the scale, the more perseverance that person possesses.

#### Ten item personality inventory

The TIPI was developed based on the Big-Five framework, and has been successfully tested and validated among elementary school students [17] as well as university undergraduates [41]. The scale still comprises five subscales: Extraversion, Agreeableness, Conscientiousness, Emotional Stability, Openness to Experience. This scale consists of 10 items, with each facet assessed by 2 items on a 7-point Likert scale ranging from 1 (*strongly disagree*) to 7 (*strongly agree*). The test-retest coefficient was 0.72.

#### Self-control scale

The scale for measuring self-control was revised by Unger and his colleagues [42]. The SCS includes 13 items, and all items were rated on a five-point Likert scale ranging from 1 (*strongly disagree*) to 5 (*strongly agree*) [42]. The Cronbach’s alpha for this scale was 0.75.

#### Effort regulation scale

To measure students’ ability to control their effort and attention when faced with boring tasks, we used the scale of the ERS, which includes 4 items and uses a 7-point Likert scale ranging from 1 (*not at all true of me*) to 7 (*very true of me*). The ERS is a subscale of the Motivated Strategies for Learning Questionnaire [43]. The Cronbach’s alpha for this scale was 0.69.

#### Academic achievement

To evaluate students’ academic achievement, we used reading and mathematics tests. The test proficiency values *θ* was calculated by using a model-based scaling procedure based on Item Response Theory (IRT) that applied to dichotomous or more graded responses to testing items [44, 45]. Next, the proficiency values θ was transformed into a scale score with a mean of 500 and a standard deviation of 100 (T = 500 + 100*ability).

### Statistical analysis

Firstly, the item discrimination of the GS-SC was tested using the critical ratio (CR), with the value of CR exceeding 3 is considered acceptable [46–48]. Pearson correlation analysis was also used to examine the correlation between the items and the total score, with a value over 0.4 as standard [49].

Secondly, factor analysis including exploratory factor analysis (EFA) and confirmatory factor analysis (CFA) were used to test the construct validity of the GS-SC. Sample 1 (*n* = 701) was used for EFA. The measure of sampling adequacy (MSA) and Bartlett’s test of sphericity were used to assess sampling adequacy and the appropriateness of data for performing factor analysis, respectively. Maximum likelihood estimation was used as the factor extraction method, and a promax (oblique) rotation was utilized given the interrelation between two dimensions of GS-SC items. The parallel analysis and scree test were used to identify the optimum number of factors to extract.

Sample 2 (*n* = 5384) was used for CFA to verify the factor structure of the GS-SC derived from EFA. To examine the two-factor structure models, CFA was conducted using Maximum Likelihood estimation. Given that in large sample sizes the *χ*^2^ statistic is likely to be significant, the normed fit index (NFI), the Tacker-Lewis index (TLI), the comparative fit index (CFI), the goodness-of-fit index (GFI), the root mean square error of approximation (RMSEA) and root mean square residual (RMR) were calculated to evaluate the model fit in this study. Values of NFI, TLI, CFI, and GFI greater than 0.90 indicate an acceptable fit [50, 51]. An RMSEA value below 0.06 and an RMR value below 0.08 indicate a relatively acceptable fit [52]. We also examined the average variance extracted (AVE) to further evaluate the convergent validity and discriminant validity of the GS-SC. An AVE of 0.5 or higher suggests sufficient convergent validity [53]. AVE estimates for two factors should also be greater than the square of the correlation between the two factors to provide evidence of discriminant validity [53]. A composite reliability of 0.7 or higher is deemed sufficient for indicating convergence or internal consistency [53].

Thirdly, the internal consistency reliability of the GS-SC was evaluated with McDonald’s ω. The value of McDonald’s ω reflects the similarity among all items, with higher value indicating that the total score of the scale more reliable [54, 55]. It was suggested that McDonald’s ω values should exceed 0.50, and a value of 0.75 or higher would be preferred [56]. Following the finalization of the GS-SC, bivariate correlations and descriptive statistics were obtained for all study variables.

Fourthly, four different forms of measurement invariance across gender were examined by employing a multi-group CFA model: configural invariance(identical factor structure), metric invariance(factor loadings were set to be equal across gender), scalar invariance(factor loadings and intercepts were constrained equal across gender), and error variance invariance(factor loadings, intercepts, and error variance were constrained equal across gender) [57]. Following previous recommendations, a decrease in CFA of ≥ 0.01, and an increase in RMSEA of ≥ 0.015 was taken as an unacceptable decrease of model fit, which means that measurement variance could not be established [58–60].

Lastly, with applying a multidimensional graded response model (GRM), an additional test based on Item response theory were conducted, supplementing additional examinations about psychological properties of the GS-SC. Relevant indicators included discrimination, difficulty, test information, and Item characteristic curve(ICC). It is acceptable when values of discrimination parameters are above 1 and common range of threshold value is -4 to 4 [61, 62]. Item fit statistics were checked via the Orlando and Thissen signed chi-squared test (S-*χ*^2^) [63]. Meanwhile, RMSEA values less than 0.08 indicates that an item fits the multidimensional GRM.

Statistical analyses were conducted with JASP 0.14.1.0 and R Studio for Windows (Version 1.1.463).

## Results

### Item analysis

The mean scores, skewness, kurtosis, C.R. and item-total correlations (ITC) of each item for Sample 1 were showed in Table 1. As shown in the table below, all the skewness and kurtosis statistics were acceptable, indicating that the data were normally distributed [64]. all of the CR values obtained for the GS-SC, ranging from 14.08 to 19.91, which reached statistical significance (*p* < .001), implying that the 11 items in the GS-SC exhibited good discriminatory power [46–48]. The item-total correlation coefficients of all items were higher than 0.4 ranging from 0.48 to 0.60, indicating that the item content could reflect typical behaviors of the grit trait.

**Table 1:** Mean scores, standard deviation, measures of distribution and the item-total correlation of the GS-SC

**Item**	**Sample 1** (*n* = 701)						**Sample 2** (*n* = 5384)				
	**Mean**	**SD**	**Skewness**	**Kurtosis**	**C.R.**	**ITC**	**Mean**		**SD**	**Skewness**	**Kurtosis**
G1	3.31	1.39	− 0.36	-1.11	19.91^***^	0.57	3.50		1.41	− 0.55	-1.00
G3	3.04	1.45	− 0.11	-1.37	18.21^***^	0.51	3.10		1.46	− 0.15	-1.34
G4	3.33	1.39	− 0.38	-1.10	18.16^***^	0.53	3.56		1.39	− 0.60	− 0.91
G5	2.85	1.48	0.07	-1.42	17.39^***^	0.49	2.93		1.50	0.03	-1.44
G6	3.35	1.33	− 0.46	− 0.87	17.82^***^	0.55	3.56		1.34	− 0.60	− 0.79
G7	2.79	1.43	0.16	-1.29	18.70^***^	0.55	2.86		1.45	0.11	-1.33
G8	2.76	1.37	0.18	-1.17	14.53^***^	0.48	2.98		1.42	− 0.01	-1.29
G9	3.49	1.28	− 0.55	− 0.73	18.96^***^	0.60	3.69		1.28	− 0.73	− 0.51
G10	3.55	1.24	− 0.56	− 0.57	17.33^***^	0.57	3.72		1.28	− 0.77	− 0.46
G11	2.93	1.46	0.03	-1.35	17.51^***^	0.49	2.96		1.50	0.02	-1.41
G12	3.51	1.26	− 0.52	− 0.68	14.08^***^	0.49	3.62		1.25	− 0.58	− 0.65

* *p* < .05, ** *p* < .01, *** *p* < .001

### Exploratory factor analysis

The overall MSA value was 0.86 and the MSA values for each item were all above 0.70. Bartlett’s test of sphericity (*p* < .001) indicated that significant correlations existed among the items, allowing for further analysis. Thus, the results showed that data of the GS-SC is suitable for exploratory factor analysis.

**Fig. 1:**
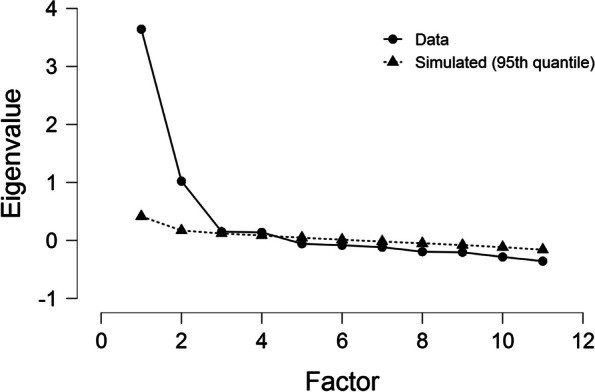
Parallel analysis and scree plot of the EFA of the GS-SC items (*N* = 701)

Next, we conducted a parallel analysis and a scree test to explore the optimum number of factors to retain. As shown in Fig. 1, the scree plot showed an obvious flattening point beginning at Factor 3. A commonly used criterion suggests that the number of factors to retain is one less than the factor number of the flattening point [65]. The parallel analysis indicated two factors, as the mean of the eigenvalues in parallel analysis exceeded the actual eigenvalue. Based on the results of the parallel analysis and the scree test, we decided to retain two factors.

The first factor had an eigenvalue of 4.40 (accounts for 39.96% of the variance), while the second had an eigenvalue of 1.69 (accounts for 15.40% of the variance). Together, these two factors solution account for 55.36% of the cumulative variance of the 11 items, deemed sufficient in terms of total variance explained. Extracted communalities of the items ranged between 0.43 (G4) and 0.69 (G7), showing that all items had moderate loadings in the GS-SC (Table 2), given that loadings above 0.40 are acceptable [53]. The factor loadings for perseverance of effort ranged from 0.64 to 0.78, while the factor loadings for consistency of interest 0.67 to 0.83.

**Table 2:** Item Properties and EFA factor loadings of the GS-SC with promax rotation

**Item**	**Perseverance of Effort**	**Consistency of Interest**	**Communalities**
G10.为了达成目标, 在过程里我会很努力地去准备与投入。 I have achieved a goal that took years of work.	0.78		0.60
G6.我是个努力的人。 I am a hard worker.	0.77		0.60
G9.我做事有始有终。 I finish whatever I begin.	0.77		0.60
G12.我很勤奋。 I am diligent.	0.75		0.58
G1.我曾经为了完成重要挑战而去克服挫折。 I have overcome setbacks to conquer an important challenge.	0.75		0.56
G4.我不会因为挫折就气馁。 Setbacks don’t discourage me.	0.64		0.43
G7.我经常设定一个目标后不久又改追求另一个目标。 I often set a goal but later choose to pursue a different one.		0.83	0.69
G5.我会短暂地着迷于某个想法或计划, 但不久后就失去兴趣。 I have been obsessed with a certain idea or project for a short time but later lost interest.		0.79	0.64
G3.我的喜好时常在改变。 My interests change from year to year.		0.68	0.47
G11.我每隔一阵子就会去追求新的目标。 I become interested in new pursuits every few months.		0.68	0.46
G8.把注意力集中在花好几个月才能完成的计划上, 对我来说是困难的。 I have difficulty maintaining my focus on projects that take more than a few months to complete.		0.67	0.46
Extraction Sums of Squared Loadings	4.40	1.69	
variance	39.96	15.40	
cumulative variance	39.96	55.36	

The reliability of the GS-SC is shown in Table 3. The McDonald’s ω values in the current study were 0.87 and 0.83 for perseverance of effort and consistency of interest, respectively. These values demonstrate that the GS-SC exhibit good internal consistency.

**Table 3:** Reliabilities of the GS-SC

Factor	Item	McDonald’s ω
Perseverance of Effort	6	0.87
Consistency of Interest	5	0.83

**Fig. 2:**
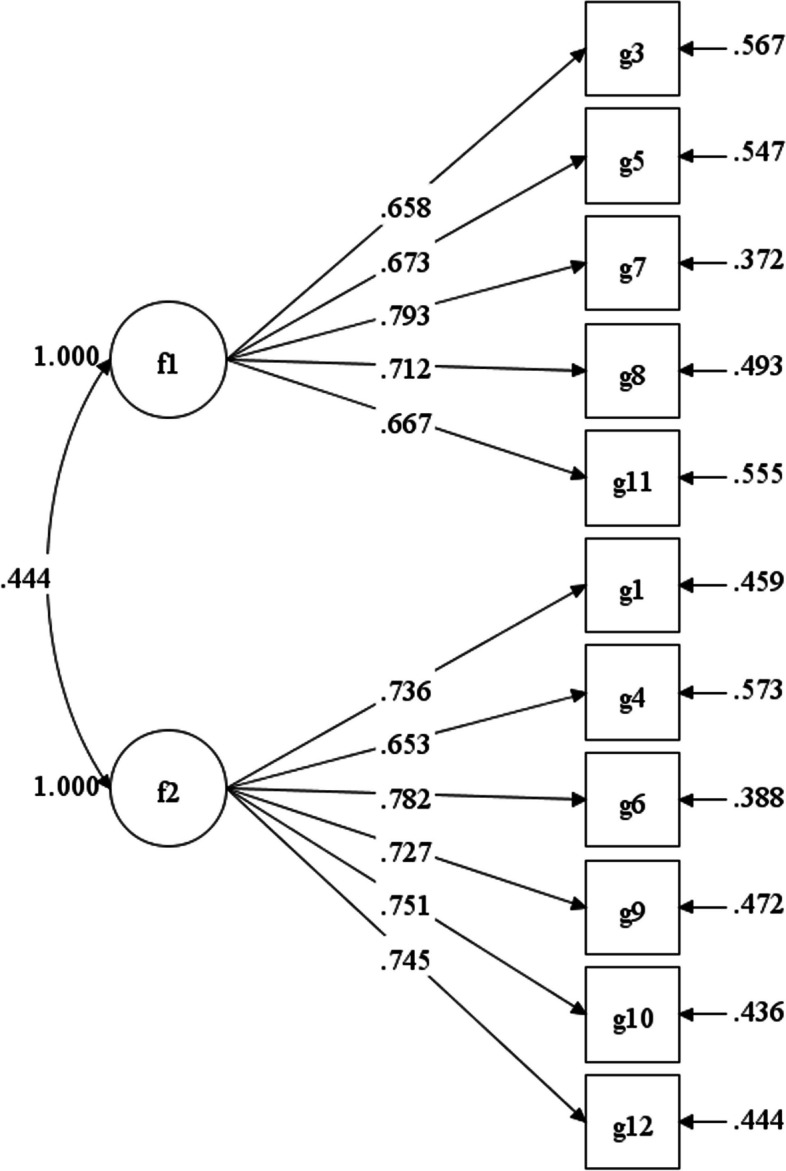
Higher order two-dimensional model of the GS-SC

The fit of the model 1 [3], model 2 [17] and model 3 for the two-factor model are reported in Table 4. Model 1 denotes the confirmatory factor analysis (CFA) results from the Grit-O [3], Model 2 denotes the results from the GS-TC [17], and Model 3 denotes the results from this study’s CFA(Fig. 2). Results of the model 1 suggested barely adequate fit in the original sample [3]. In contrast, as illustrated in Table 4, a good fit was found for the model 3, comprising of the model 1 and model 2.

**Table 4:** Summary of CFA results of the different GS models

Model	χ ^2^/df	RMSEA	SRMR	GFI	NFI	TLI	CFI
Model 1	56.94	0.10	0.09	0.91	0.89	0.87	0.89
Model 2	3.22	0.07	0.08	0.99	0.80	0.88	0.88
Model 3	25.72	0.07	0.04	0.96	0.96	0.94	0.96

The composite reliability (CR) for the Perseverance of Effort was 0.88, whereas the CR of Consistency of Interest was 0.83. Both values exceed the suggested threshold of 0.70, indicating adequate reliability [53]. The AVEs were 0.54 and 0.49 for perseverance of effort and consistency of interest, respectively. The correlation between Perseverance of Effort and Consistency of Interest was 0.40, and its squared value was 0.16. Thus, discriminant validity between the two factors is supported because the AVE of each factor was greater than the squared correlation between them [53].

Model 1 is the CFA results of the Grit-O [3], Model 2 is the CFA results of the GS-TC [17], and Model 3 is the fits of 11-item version of grit scale in the study. Specifically, Model 1 consists of two dimensions (Consistency of Interests and Perseverance of Effort), with each dimension containing 6 items. Model 2 was similar to Model 1, but in the Consistency of Interests dimension, one item (g2) was removed due to its low correlation with other items and difficulty in comprehension [17]. The structure of Model 3 was identical with that of Model 2.

### Criterion Validity

The correlations between the GS-SC and the four other measures are shown in Table 5. The results showed that grit was positively correlated with self-control (*r* = .51, *p* < .001), effort regulation (*r* = .46, *p* < .001), extraversion (*r* = .24, *p* < .001), agreeableness (*r* = .37, *p* < .001), conscientiousness (*r* = .35, *p* < .001), emotional Stability (*r* = .35, *p* < .001), openness (*r* = .37, *p* < .001), reading(*r* = .30, *p* < .001), and mathematics (*r* = .26, *p* < .01).

Perseverance of effort was negatively correlated with consistency of interest (*r* =- .40, *p* < .001). Perseverance of effort was positively correlated with self-control (*r* = .28, *p* < .001), effort regulation (*r* = .41, *p* < .001), extraversion (*r* = .18, *p* < .001), agreeableness (*r* = .25, *p* < .001), conscientiousness (*r* = .26, *p* < .001), emotional Stability (*r* = .24, *p* < .001), openness (*r* = .29, *p* < .001), reading (*r* = .25, *p* < .001) and mathematics (*r* = .23, *p* < .01). Consistency of interest was positively correlated with self-control (*r* = .28, *p* < .001), effort regulation (*r* = .08, *p* < .001), extraversion (*r* = .08, *p* < .001), agreeableness (*r* = .16, *p* < .001), conscientiousness (*r* = .13, *p* < .001), emotional Stability (*r* = .13, *p* < .001), openness (*r* = .10, *p* < .001), reading(*r* = .07, *p* < .001) and mathematics (*r* = .06, *p* < .001).

**Table 5:** Correlation among grit, self-control, effort regulation, big-five personality, reading and mathematics

**Variable**	**M**	**SD**	**1**	**2**	**3**	**4**	**5**	**6**	**7**	**8**	**9**	**10**	**11**	**12**
1.grit	3.36	0.59	—											
2.Perseverance of effort	3.63	1.03	0.61^***^	—										
3.Consistency of interest	3.03	1.13	0.49^***^	− 0.40^***^	—									
4.Self-control	43.59	8.35	0.51^***^	0.28^***^	0.28^***^	—								
5.Effort regulation	19.29	3.51	0.46^***^	0.41^***^	0.08^***^	0.52^***^	—							
6.Extraversion	8.64	2.74	0.24^***^	0.18^***^	0.08^***^	0.21^***^	0.19^***^	—						
7.Agreeableness	9.50	2.69	0.37^***^	0.25^***^	0.16^***^	0.38^***^	0.32^***^	0.05^**^	—					
8.Conscientiousness	8.76	2.80	0.35^***^	0.26^***^	0.13^***^	0.38^***^	0.30^***^	0.24^***^	0.17^***^	—				
9.Emotional Stability	9.12	2.81	0.35^***^	0.24^***^	0.13^***^	0.37^***^	0.30^***^	0.17^***^	0.34^***^	0.27^***^	—			
10.Openness	9.09	2.80	0.37^***^	0.29^***^	0.10^***^	0.36^***^	0.32^***^	0.20^***^	0.26^***^	0.36^***^	0.19^***^	—		
11.reading	508.19	49.19	0.30^***^	0.25^***^	0.07^***^	0.27^***^	0.27^***^	0.18^***^	0.17^***^	0.21^***^	0.15^***^	0.19^***^	—	
12.mathematics	502.67	49.47	0.26^***^	0.23^***^	0.06^***^	0.23^***^	0.23^***^	0.15^***^	0.14^***^	0.19^***^	0.13^***^	0.18^***^	0.65^***^	—

### Test-retest reliability

We calculated the Pearson’s correlation coefficients to verify test-retest reliability of the overall GS-SC and each factor. The results indicated that the GS-SC (*r* = .64, *p* < .001), perseverance of effort (*r* = .55, *p* < .001), and the consistency of interest (*r* = .66, *p* < .001) exhibited adequate test-retest reliability, suggesting that the GS-SC is a stable instrument for measuring the character strength of grit.

### Gender differences

Before the examination of possible gender difference, the measurement invariance was test first. As shown in Table 6, the change of CFI and RMSEA values between models (Metric vs. Configural, Scalar vs. Metric, and Error variance vs. Scalar) were all less than 0.01, indicating the scale possessed strict measurement invariance and it is meaningful to employ its scores for the following intergroup comparisons.

**Table 6:** Model fit for multiple group models and measurement invariance comparisons

Models	χ ^2^	df	CFI	RMSEA [90% CI]
Configural invariance	762.992	88	0.960	0.054[0.050, 0.057]
Metric invariance	803.363	97	0.959	0.053[0.049, 0.056]
Scalar invariance	883.328	108	0.955	0.052[0.049, 0.055]
Error variance invariance	907.065	119	0.954	0.050[0.047, 0.053]

As shown in Table 7, the consistency of interest scores of the boys were significantly lower than those of the girls (*t* = 3.27, *p* < .01, *d* = 0.09). The GS-SC scores of boys were significantly lower than those of the girls (*t* = 3.63, *p* < .001, *d* = 0.11). However, there were no significant differences found between girls and boys for the perseverance of effort (*t* = 0.89, *p* > .05, *d* = 0.03).

**Table 7:** Mean, standard deviation and t-test between the girls and the boys

variables	Boys			Girls			*** t ***	*** d ***
	**N**	**M **	** SD **	**N**	** M **	** SD **		
Perseverance of effort	2643	3.62	1.08	2201	3.65	0.98	0.89	0.03
Consistency of interest	2681	2.98	1.15	2238	3.09	1.09	3.27^**^	0.09
Average of GS-SC	2643	3.33	0.58	2201	3.39	0.60	3.63^***^	0.11

### MIRT analysis

The fit for the multidimensional GRM was acceptable (*χ*^2^ = 293.55, *df* = 10, *p* < .001, CFI = 0.93, TLI = 0.85, SRMR = 0.10, RMSEA = 0.07). Table 8 shows the item parameter estimated via the GRM. All the items display an excellent fit with the GRM model. The discrimination parameters estimated ranged from 2.13 to 3.45, which were all considered very high [66]. Furthermore, all the GS-SC items reported difficulty thresholds that proceeded from less to more difficulty, thus well reflecting the ordered categorical feature of the 5-point Likert scale. Among all the threshold parameters, only the fourth threshold values were positive, and all of the first and second thresholds were negative. It indicates that most of the items in GS-SC are relatively easy, which means it is possible for students with average level of grit could get a relatively high score on this scale.

**Table 8:** Multidimensional item response theory (MIRT) analyses

Item	S_χ^2^	df.S_χ^2^	RMSEA.S_χ^2^	*p*.S_χ^2^	a(1)	a(2)	b1	b2	b3	b4
a1	1407.29	116	0.048	0.00	2.86		-1.19	− 0.82	− 0.31	0.40
a4	1375.98	121	0.046	0.00	2.33		-1.37	− 0.93	− 0.41	0.40
a6	1311.25	112	0.047	0.00	3.38		-1.28	− 0.88	− 0.32	0.42
a9	1279.65	116	0.045	0.00	2.85		-1.48	-1.04	− 0.44	0.36
a10	1367.91	113	0.048	0.00	3.18		-1.41	-1.02	− 0.44	0.30
a12	1274.37	114	0.046	0.00	2.88		-1.58	-1.00	− 0.31	0.46
a3	1627.98	126	0.050	0.00		2.13	− 0.98	− 0.44	0.13	0.88
a5	2020.27	126	0.056	0.00		2.21	− 0.77	− 0.27	0.25	0.93
a7	1983.01	111	0.059	0.00		3.45	− 0.69	− 0.23	0.29	0.92
a8	2025.77	122	0.057	0.00		2.51	− 0.90	− 0.36	0.26	0.95
a11	1918.95	126	0.054	0.00		2.21	− 0.81	− 0.29	0.27	0.90

**Fig. 3:**
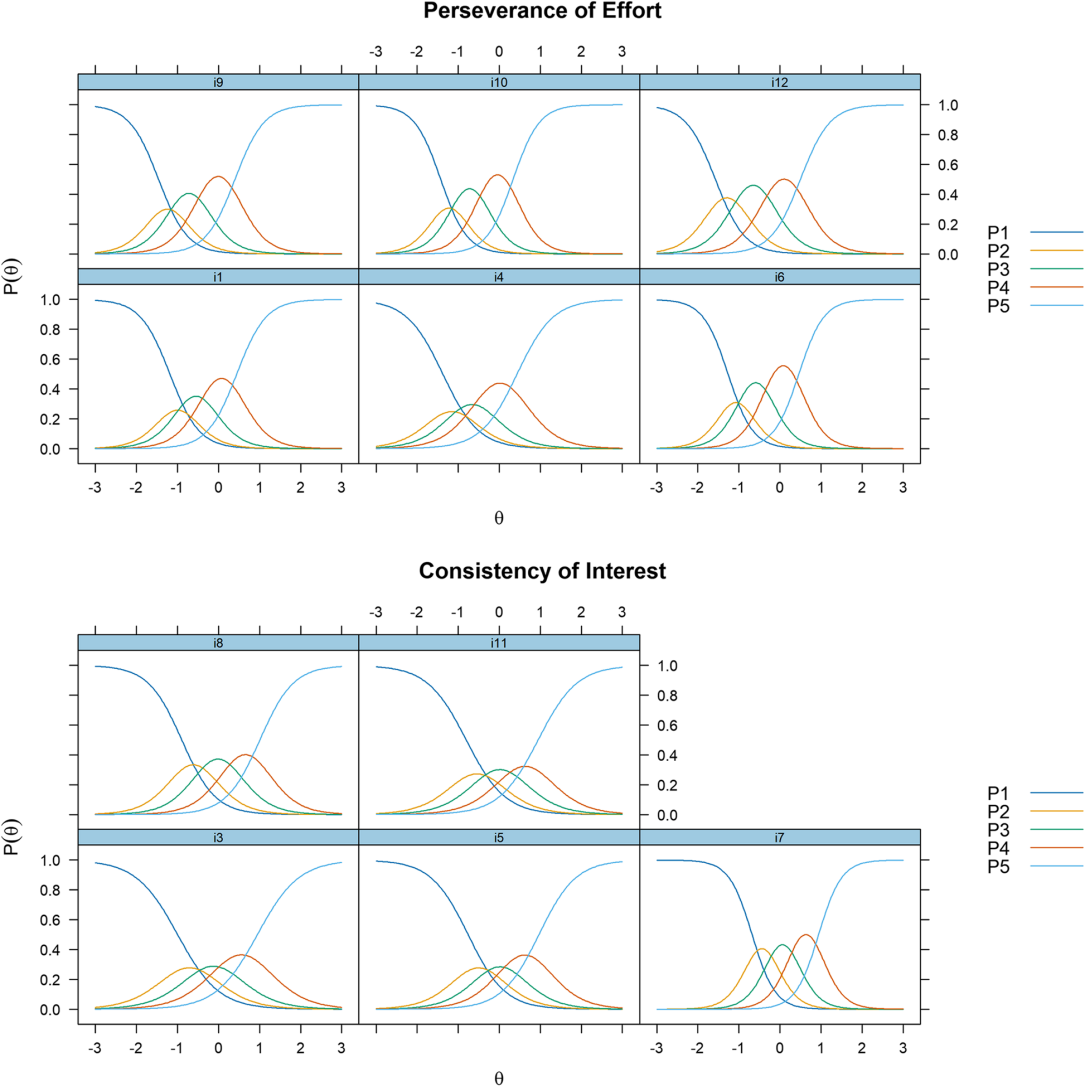
Item characteristic curves for the Perseverance of Effort and Consistency of Interest subscales for the GS-SC was displayed above, respectively. For each graph, the x axis demonstrates the distribution of latent trait, while the y axis demonstrates the probabilities of students choosing specific options

Lastly, Fig. 3 shows the item characteristic curves for GS-SC-11 items. It is shown that for most items, the distribution of P2, P3, P4 (corresponding with options scoring 2–4 in the scale) is relatively compact. It means that discriminative ability of these three options were comparatively lower than others. Namely, for students whose levels of grit trait was as at the middle of the trait distribution, these three options do not exhibit significant difference.

## Discussion

The primary aim of this study was to examine the psychometric properties of the GS-SC among a large sample of children in the mainland of China. After removing “G2. New ideas and new projects sometimes distract me from previous ones”, the results indicated that the GS-SC has appropriate psychometric properties and is a relatively reliable and valid instrument for measuring the grit in Chinese children. Consistent with the widely recognized theoretical framework, the GS-SC developed in this study has two dimensions: consistency of interest and perseverance of effort [17]. Our findings also offered validity evidence of the connections between the GS-SC scores and other metrics.

The second question of the GS-SC was found to be weakly connected with several items on the interest subscale, according to the results of the correlation analysis. Additionally, in the follow-up interviews, it was discovered that some kids had trouble understanding the meaning of the second question, which might have affected their responses. Besides, previous study [17] have found that the second question is not suitable for primary school students, consistent with the results of this study. Taking these considerations into account, the researcher chose to remove the second question. There can be cultural differences between China and the West on the second question of the grit scale. For Chinese elementary school students, providing an answer to this question is difficult.

To examine evidence for the criterion-related validity of the GS-SC, the associations of the grit with self-control, effort regulation, personality, academic achievement (reading and mathematics) were evaluated. In prior studies, grit has demonstrated a positive association with self-control [3, 17, 23, 27]. As hypothesized, we found that grit exhibited stronger relations with self-control. The correlation coefficient between self-control and the perseverance of effort is the same as the correlation coefficient for the consistency of interest.

Grit is not only related to the big five personality trait of conscientiousness, but also positively related to the other four variables. Our results regarding the relationship between grit and the big five personality were inconsistent with some of the prior research [17, 67], but were similar to the results of Duckworth, Peterson, Matthews and Kelly [3]. Although both the perseverance of effort and the consistency of interest significantly correlated with the big five personality, the correlation coefficients for the perseverance of effort were larger than those for the consistency of interest. Moreover, scores for the traits openness and agreeableness were low, which was consistent with results of previous meta-analyses [27].

Both reading and mathematics scores were significantly correlated with the total score of the GS-SC, indicating that our results regarding the relationship between grit and academic achievement is consistent with similar observations among Western and Eastern participants [3, 17, 18, 36]. Overall, these findings suggest that the impact of grit on academic success may be universal across cultures. However, while the total score of the GS-SC correlated significantly with academic achievement, its two dimensions did not consistently correlate with academic achievement. The correlation coefficient between the perseverance of effort and reading and mathematics was higher than that for the consistency of interest. This result is consistent with the findings of previous meta-analyses [27, 35]. Therefore, compared to CI, PE was a stronger predictor of academic success and retention.

There was significant difference in grit score between gender, with girls scoring slightly higher than boys. This result did not support findings of Tsai, Lin, Chen, Lin and Tsai [17], who reported that there might be no difference on the levels of grit between boys and girls. However, the result was congruent with the study of Sigmundsson, Guðnason and Jóhannsdóttir [68], which also found that women were slightly more likely than men to have higher grit scores. In a study focused on the elementary students in the US, Christensen and Knezek [69] also found significant differences between females and males using the GS-O, with female students had higher scores. Although girls in this study had significantly higher grit score than boys, the effect sizes was small. It implies that the gap between the two may not be very different, or that females may be slightly more likely than males to have higher scores on certain grit scales and in various cultural situations.

The current study had several potential limitations. Although our sample size was large, all the participants were recruited solely from Zhejiang province, which is known for having higher quality of education compared to other regions in western and central China. Thus, our samples might not be sufficiently representative of the larger population.

## Conclusion

This study’s findings suggested that the GS-SC demonstrated acceptable psychometric properties, providing accurate measurements of grit and making it a suitable instrument for measuring the grit levels among elementary school students. Furthermore, the current study provides further validation that it is necessary to remove second item G2 to ensure the instrument’s reliability and validity in the background of Chinese culture.

## Data Availability

All data that are related to the findings of this study are available from the corresponding author upon reasonable request.
